# An Improved Otsu Threshold Segmentation Method for Underwater Simultaneous Localization and Mapping-Based Navigation

**DOI:** 10.3390/s16071148

**Published:** 2016-07-22

**Authors:** Xin Yuan, José-Fernán Martínez, Martina Eckert, Lourdes López-Santidrián

**Affiliations:** Centro de Investigación en Tecnologías Software y Sistemas Multimedia para la Sostenibilidad (CITSEM), Campus Sur, Universidad Politécnica de Madrid (UPM), Madrid 28031, Spain; jf.martinez@upm.es (J.-F.M.); martina.eckert@upm.es (M.E.); lourdes.lopez@upm.es (L.L.-S.)

**Keywords:** threshold segmentation, underwater object detection, simultaneous localization and mapping (SLAM), augmented extended Kalman filter (AEKF)

## Abstract

The main focus of this paper is on extracting features with SOund Navigation And Ranging (SONAR) sensing for further underwater landmark-based Simultaneous Localization and Mapping (SLAM). According to the characteristics of sonar images, in this paper, an improved Otsu threshold segmentation method (TSM) has been developed for feature detection. In combination with a contour detection algorithm, the foreground objects, although presenting different feature shapes, are separated much faster and more precisely than by other segmentation methods. Tests have been made with side-scan sonar (SSS) and forward-looking sonar (FLS) images in comparison with other four TSMs, namely the traditional Otsu method, the local TSM, the iterative TSM and the maximum entropy TSM. For all the sonar images presented in this work, the computational time of the improved Otsu TSM is much lower than that of the maximum entropy TSM, which achieves the highest segmentation precision among the four above mentioned TSMs. As a result of the segmentations, the centroids of the main extracted regions have been computed to represent point landmarks which can be used for navigation, e.g., with the help of an Augmented Extended Kalman Filter (AEKF)-based SLAM algorithm. The AEKF-SLAM approach is a recursive and iterative estimation-update process, which besides a prediction and an update stage (as in classical Extended Kalman Filter (EKF)), includes an augmentation stage. During navigation, the robot localizes the centroids of different segments of features in sonar images, which are detected by our improved Otsu TSM, as point landmarks. Using them with the AEKF achieves more accurate and robust estimations of the robot pose and the landmark positions, than with those detected by the maximum entropy TSM. Together with the landmarks identified by the proposed segmentation algorithm, the AEKF-SLAM has achieved reliable detection of cycles in the map and consistent map update on loop closure, which is shown in simulated experiments.

## 1. Introduction

In recent years, underwater vehicles are increasingly being used in complex environments like seas, harbors or dams, and underwater robotic mapping has received considerable attention from the research community. Robotic mapping is the process of generating a spatial representation of a given environment from a series of sensor measurements observed by the robot while travelling through that environment. An accurate map is a fundamental and mandatory requirement for a robot to work autonomously. The use of the conventional Kalman filter (KF) method is not adequate for estimating underwater landmark positions, as it suffers from the assumption of Gaussian noise statistics, which often lead to failures when these assumptions do not hold. Additionally, the linearization errors caused by the standard extended Kalman filter (EKF) can also severely degrade the performance of the localization estimates. This paper comes up with two proposals and their combination: the first one is an improved Otsu Threshold Segmentation Method (TSM), which provides a real time and accurate segmentation solution for underwater feature detection and has been proved to be faster than other TSMs. The second one is an augmented EKF (AEKF)-based underwater landmark SLAM, which employs the landmarks detected by the improved Otsu TSM. The SLAM algorithm jointly estimates the geometric landmark locations and the vehicle pose with the help of a stochastic framework. Underwater SLAM is a tough variable dimension state estimation problem in which the size of the state space is increased or decreased when features are added or removed from the map. While the robot moves through the underwater environment, it is exploring the region without having exact geo-referencing data, so it uses sensor measurements to perform two basic operations: one is updating concurrently its own state estimate and refining the previously observed landmark positions in the environment, the other is adding newly detected features into the state of the overall system.

The study of estimation-theoretic solutions for the SLAM problem within the field of robotics has received considerable attention within the research community. Initial works by Smith et al. [1] and Durrant-Whyte [2] established a statistical basis for representing relationships between landmarks and manipulating geometric uncertainty. Meanwhile, Ayache and Faugeras [3] and Chatila and Laumond [4] were working in the visual navigation of mobile robots using KF-based algorithms. Then, Smith, Self and Cheeseman [5] proved that, when a mobile robot moves through an unknown environment measuring relative observations of landmarks, the estimates of these landmarks are all necessarily correlated with each other, due to the common error in the estimated robot pose. The SLAM problem can be stated from different approaches depending on the number of landmarks, the area of coverage, computational requirements, flexibility, etc. [6]. Recently, probabilistic approaches such as KF, Particle Filters (PF) and Expectation Maximization (EM) have become dominant methods to deal with the stochastic SLAM problem. These three techniques are mathematical derivations of the recursive Bayes rule. Stochastic SLAM suffers from three main weaknesses: high computation and storage costs, fragile data association, and inconsistent treatment of non-linearity. High computation and storage are the price paid for maintaining correlations in the system state covariance matrix.

At present, there exist robust methods for mapping environments that are static, structured, and of limited size. These methods deal with issues related to computational complexity, data association and representation of the environment [7,8]. However, the implementation of SLAM in real underwater environments still can be considered as an unsolved and challenging research topic due to the limitations of subsea localization and perception sensors followed by error accumulation over long-term operations. Furthermore, sonar measurements are affected by multiple sources of perturbations such as hydrostatic effects of waves or currents, inhomogeneous pressure distribution, marine animals, vessel traffic and the vessel propellers. Besides, one needs to deal with reflections and poor resolution of the acoustic imaging for extracting features. Although video cameras provide highly detailed images, they are always limited due to turbidity and low-lighting conditions [9]. Moreover, the dynamic continuous changes of underwater natural resources is another factor that makes the recognition of previously visited locations and identification of already detected features difficult or even impossible. Today large scale unstructured and dynamic environments are still presenting multiple challenges for SLAM applications.

The next section of this paper presents the related works about the state of the art of the underwater SLAM problem, and three kinds of currently used maps in mobile robot navigation systems are compared, being the landmark map the most suitable one to represent the undersea environment. Section 3 describes the improved Otsu TSM, and compares its segmentation accuracies and computational costs of a shipwreck, a branch from two side-scan sonar (SSS) images and a plastic mannequin from a forward-looking sonar (FLS) imagery with the ones of the traditional Otsu, the local TSM, the iterative TSM and the maximum entropy TSM, respectively. Also, the centroids of different detected regions have been calculated, which are used as point landmark positions in the MATLAB simulation experiment of AEKF-based SLAM loop mapping in Section 4. Furthermore, Section 4 reviews the mathematical model of the estimation-theoretic AEKF-based underwater landmark SLAM approach. Section 5 concludes this paper and discusses the future work of implementing the AEKF algorithm in combination with the proposed improved Otsu TSM to solve large-scale underwater SLAM problem.

## 2. Related Works

At present, underwater mapping techniques put great emphases on the integration of data recorded from different perception and navigation sensors, generally acoustic sonars are used to create large scale maps of the environments while optical cameras provide more detailed images of the objects of interest. Since the costs of offshore seabed mapping are largely determined by the expense of the vehicle exploring time, any improvement in the quality of the sonar perceived data, and especially reduction in detection costs are of crucial interest to the marine and offshore community. In this work, the proposed segmentation method achieves both a much lower False Positive Rate (FPR) and computational time, also a much higher precision rate than those values of the maximum entropy TSM, which has the highest segmentation precision among the four classic methods compared in this work.

In the SLAM context, features must be distinguishable enough to simplify the data association process of new observations to corresponding map features. In general, features can be detected by their characteristics, such as positions, shapes, and colors. In underwater environments, there should be as many features as possible, which could be observed repeatedly, so as to reduce the uncertainty caused by significant vehicle drift. Currently, there exist several feature extraction approaches. Edge, contour and corner detectors are commonly used in computer vision, for instance the well-known Canny edge detector [10], and the Harris corner detector [11]. Different invariant solutions, such as Scale-Invariant Feature Transform (SIFT) [12] and Speeded Up Robust Features (SURF) [13] produce more robust, rotation and scale invariant features. 

Problems related to underwater SLAM are nowadays a hot topic in the robotics community. Recent works such as [14] present a probabilistic underwater SLAM using a multibeam echosounder to generate 3D high consistency bathymetry maps of the inspected region in the form of point cloud data. The authors come up with point-to-point and point-to-plane associations for registering the point clouds, and they use two real world datasets to test their algorithm, one is a 2.5D bathymetric dataset, the other is a full 3D underwater dataset. As a result, the consistency of their proposed SLAM framework is higher than that acquired with dead reckoning. Since high-quality images could help the data association process, a great number of research papers focus on image segmentation, classification, registration and landmark extraction. Other papers address the issue of data association in the context of sonar images [15,16] and some work on matching side-scan sonar images [17]. In [18], the systematic geometrical distortions of the SSS maps are corrected and the authors achieve an underwater maps mosaic of the surveyed scenario by using a multibeam FLS. Very few references can be found regarding the use of Fourier based registration approaches for sonar imagery and [19] is the first work considering this application on 2D FLS images. Phase correlation has already been applied to register underwater optical images for building photo mosaics. This paper achieves aligning consecutive and non-consecutive sonar frames with high accuracy and robustness by using the phase correlation algorithm. In [20], a new method for registering 2D FLS images recorded from real mobile platform by optimizing over four (of the six) degrees of freedom (DOF) in the 3D sonar motion is proposed. Features and landmarks are successfully detected and effectively depicted in Gaussian maps with improved performances compared with the currently used 2D similarity transformation. Reference [21] describes FLS images considering the shape of objects and their topological relation by using a graph of Gaussian probability density function. Besides, the descriptors are matched efficiently based on similarities of the vertices of the graphs in each image. Compared with [22], which also employs the Otsu segmentation on multi-beam sonar images for feature detection, our presented work is novel in applying a power-law transform before performing different threshold segmentation methods (TSM), and it has been figured out that the best thresholding results are achieved when *y* = 0.1*x*^1.415^, in this way the contrast between object and background is enlarged and the feature is highlighted, since the gray levels of the object are increased and the background ones are decreased. Therefore, the improved Otsu TSM has more accurate segmentation performance than the methodology used in [22]. Besides, [22] does not consider the computational time of their method, but our proposed segmentation approach could keep good tradeoff between segmentation precision and computational cost. In [23], the classic Otsu thresholding and static thresholding are applied for object detection using the sector scanning sonar. Although the Otsu segmentation method requires several scanline measurements to be collated before obtaining the binary detection, the segmentation result of the Otsu approach is much cleaner than that of static thresholding, but the features which are farther away with marginal measurements and near the background noise level, cannot be detected by the classic Otsu method. The improved Otsu TSM presented in our work could solve this problem, since it is an adaptive thresholding method and it can find the best segmentation threshold of an image. The computational cost of the configuration-conjunct threshold segmentation method, described in [24], on their presented low resolution sonar image is 0.371 s, which is three times higher than that of our improved Otsu TSM (0.117 s). Also their proposed method can only extract linear objects with neat and obvious edges, not like the objects with different feature forms presented in our work. In this section, on the one hand, a brief state of the art of the underwater SLAM problem is introduced and the recent important works in the field of feature detection in sonar images are compared. On the other hand, the basic functionalities of the three most commonly used map representations are outlined and a survey is made of their suitability to a priori map localization i.e., computational complexity, reliability, etc.

### 2.1. Map Representations

Autonomous mobile robot systems rely on maps to represent the robot’s surroundings for path planning and to determine a position with respect to the environment. At present, there are three well-known map representations related to the SLAM problem: occupancy grid maps, topological maps, and landmark maps. Occupancy grid SLAM works robustly in dynamic indoor environments over a limited period of time. They are adequate for local navigation and obstacle avoidance purposes [25], so this kind of map representation is popular for describing the environment of a mobile robot, given known poses. However, as a SLAM map representation, occupancy grids do not process a proper uncertainty model, since they represent uncertainty only at a local robot-centric level, but not at a global level, they will tend to diverge in the long term operation. To adequately capture the details in more complex environments, a high resolution of cell distribution is required, which would mean a waste when areas with less complexity are treated. Techniques like quad trees or octrees [26] have been applied to reduce the space consumption problem but can also lead to increasing computational costs.

Occupancy grids maps and landmark maps are both metric maps where positions are specified in a set of Cartesian coordinates. As for topological maps, they do not rely on metric measurements and instead represent the working environment in terms of paths connected by landmark positions. Topological maps are attractive due to their efficiency and compact representation, as well as their logical organization for tasks such as fast path planning and global navigation. In our case, in undersea environments, where location recognition is more complex, there is more risk of fail to localize [27]. Since there is no explicit physical information, topological maps are not suitable for underwater SLAM applications. 

Considering the sparse spatial distribution of marine features, and the long distances between different landmarks, the metric landmark map would be more suitable to represent the underwater working environment. In general, landmark maps allow achieving a viable representation for long-term convergent SLAM in fairly small-scale exploring regions where stable landmarks are observable, computation is tractable and accumulated state uncertainty does not exceed conservative limits. These landmarks not only include the artificial landmarks put out by the researchers, but also various other seafloor features in the vicinity of the robot. The same landmark may look totally different when observed from a different view point. In this paper, the stationary points, which are the least complicated features, are considered to represent the underwater landmarks of a local area. In order to achieve a feasible and convergent operation in larger underwater spaces with moving landmarks, modifications to the basic stochastic SLAM algorithm are required.

The robot can increase the map accuracy by revisiting previously mapped regions since it can observe and correct the changes appeared to the map [28,29,30]. If it explores an environment by traversing a large cycle which is much larger than its sensing range, then identifying a return to an already mapped area is the loop closure problem, which is also known as the cycle detection and map revisiting problem. Being able to re-identify an observed feature is of importance to detect the cycle. Notice that, a closed loop can improve map consistency and localization accuracy. For this reason, it is necessary to provide the system with algorithms capable of identifying when an observation is corresponding to a new landmark or to an already seen one. Therefore, detecting and selecting proper features is a key issue to solve within underwater SLAM problems.

### 2.2. Simultaneous Localization and Mapping

Simultaneous Localization and Mapping (SLAM) or Concurrent Mapping and Localization (CML) [31,32] combines the information provided by the robot’s odometers with the Received Signal Strength Indication (RSSI) samples provided by the surrounding objects to currently track the robot’s trajectory in the environment and refine the mapping of the objects in the area [33]. In SLAM, a robot starts at an unknown place and without previous knowledge of the environment to incrementally build a consistent landmark map using its onboard sensors, while concurrently localizes itself relative to this map by keeping track of its relative position and re-observing those already detected landmarks [34,35].

After an observation, the local perception map needs to be associated with the global map to refine the robot pose and to update the environmental feature positions as well. Selecting the next best viewpoint for completing the model is a research issue by itself. The difficulty of the SLAM problem is that, in order to obtain a qualified map, a precisely estimated robot’s trajectory is required, but reducing the unbounded growing odometry errors it is needed to incorporate sensor measurements with an accurate map. SLAM presents a series of tough issues, like: (1) efficient mapping of large-scale environments; (2) correct association of sensor measurements; and (3) robust estimation of map and robot trajectory information. In this paper, the reviewed AEKF-based SLAM method contributes to each of these three areas.

## 3. An Improved Otsu TSM for Fast Feature Detection 

An automatic detection and localization of underwater objects is of great importance in Autonomous Underwater Vehicle (AUV) navigation and mapping applications. Object detection aims at separating a foreground object from the background and generating a binary image for every frame, which is of critical importance for the sonar image processing since the result will directly affect the accuracy of the following feature extraction and object localization [36]. Feature extraction is an important aspect of SLAM, in which a mobile robot with a known kinematic model, starting at an unknown location, moving through the exploring environment where contains multiple features to incrementally generate a consistent map. Geometric features, such as points, lines, circles and corners are determined as a part of the SLAM process, since these features can be used as landmarks. Generally, SLAM consists of the following parts including motion sensing, environment sensing, robot pose estimation, feature extraction and data association. The main focus of this paper is on first extracting features from underwater sonar images of different types, and then using them as landmarks for an AEKF-based underwater SLAM.

In recent years, the resolution of sonar imagery has improved significantly, such that it can be used in a much better way for further processing and analyzed with advanced digital image processing techniques. Noise filtering, radiometric corrections, contrast enhancement, deblurring through constrained iterative deconvolution, and feature extraction are usually employed to correct or to alleviate flaws in the recorded data [37]. The first step of underwater object detection is to segment the foreground features from the background. Segmentation is the process of assigning a label to every pixel in the image such that pixels with the same label share homogeneous characteristics, like color, intensity, or texture, and thereby different entities visible in the sonar imagery could be separated. A sonar image is made up of a matrix of pixels having a gray level typically on a scale from 0 to 255. The gray levels of pixels associated with foreground objects are essentially different from those belonging to the background. Normally, in typical sonar imagery, the object is composed of two parts: the highlighted areas (echo) and the shadow regions. The echo information is caused by the reflection of the emitted acoustic wave on the object while the shadow zones correspond to the areas lack of acoustic reverberation behind the object. Based on this characteristic, the threshold segmentation methods (TSM) can be used to detect the foreground object in the sonar image. In general, the Otsu method is one of the most successful adaptive methods for image thresholding [38].

### 3.1. Side-Scan Sonar Images

Under optimal conditions, side-scan sonars (SSS) can generate an almost photorealistic, two-dimensional picture of the seabed. Once several swatches are joined via mosaicing, geological and sedimentological features could be easily recognized and their interpretation would provide a valuable qualitative insight into the topography of the seabed [39]. Due to the low grazing angle of the SSS beam over the seabed, SSS provide far higher quality images than forward-looking sonars (FLS), such that the feature extraction and the data association processes will behave better. The accuracy of SLAM using SSS is more dependent on the distribution of landmarks. In general, the SSS and the multibeam FLS provide large scale maps of the seafloor that are typically processed for detecting obstacles and extracting features of interest on the areas of the seafloor [18]. 

**Figure 1:**
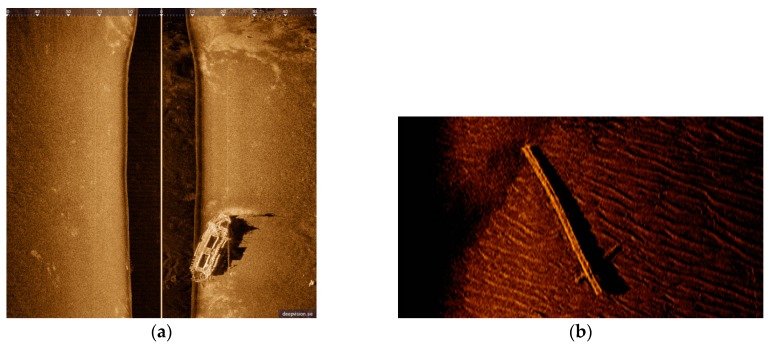
(**a**) High resolution SSS image recorded with the DE340D SSS at Stockholm sea [40]; (**b**) Low resolution SSS image generated by the 3500 Klein SSS (ECA Group company [41]).

By transmitting and receiving sound via an underwater sonar system, the seafloor terrain and texture information can be extracted with relevant data from the acoustic reflection image of the seabed. High image resolutions are very important for determining if an underwater target is something worth investigating. Two SSS images are used in this work (see Figure 1). Figure 1a is of high resolution, whereas Figure 1b is a low resolution SSS image. Actually, the DE340D SSS (Deep VisionAB company, Linköping, Sweden), the 3500 Klein SSS (Klein Marine System, Inc., Salam, MA, USA), and the Blue View P900-90 2D FLS (Teledyne BlueView, Bothell, WA, USA), all will be employed in our “Smart and Networking Underwater Robots in Cooperation Meshes”-SWARMs European project, that is the reason why we perform feature detection on these sonar images. DE340D SSS is a product of the Deep VisionAB company(Linköping, Sweden) [40], working at the frequency of 340 kHz with optimized resolution of 1.5 cm. The beam reaches from 15 to 200 m and the maximum operation depth is 100 m. The size of the converted Figure 1a is 800 × 800 = 640,000 pixels. Due to the impact of sediment and fishes, many small bright spots appear in sonar images. The area size of these background spots is usually smaller than 30 pixels, and their gray level is similar to that of certain areas of foreground object. When using the traditional TSM to separate the foreground object, in this case a shipwreck, it can be found that most of these spots are still retained in the segmentation results. To solve this problem, an improved Otsu method is proposed that constrains the search range of the ideal segmentation threshold to separate the foreground object inside the sonar imagery.

It is typical for SSS images to show a vertical white line appearing in the center, indicating the path of the sonar. A dark vertical band on both sides represents the sonar return from the water-column area. Notice that this band has no equal width, and is curved in some parts. The distance from the white line to the end of the dark area is equivalent to the depth to the sea bottom below the sonar device. The seabed is imaged on either side, corresponding to port-side and starboard-side views. Bright areas indicate ascending ground, while dark areas correspond to descending regions or shadows produced by objects, vegetation or rocks. The length of a shadow can be used to calculate the height of an object. 

### 3.2. The Proposed Improved Otsu TSM Algorithm

**Figure 2:**
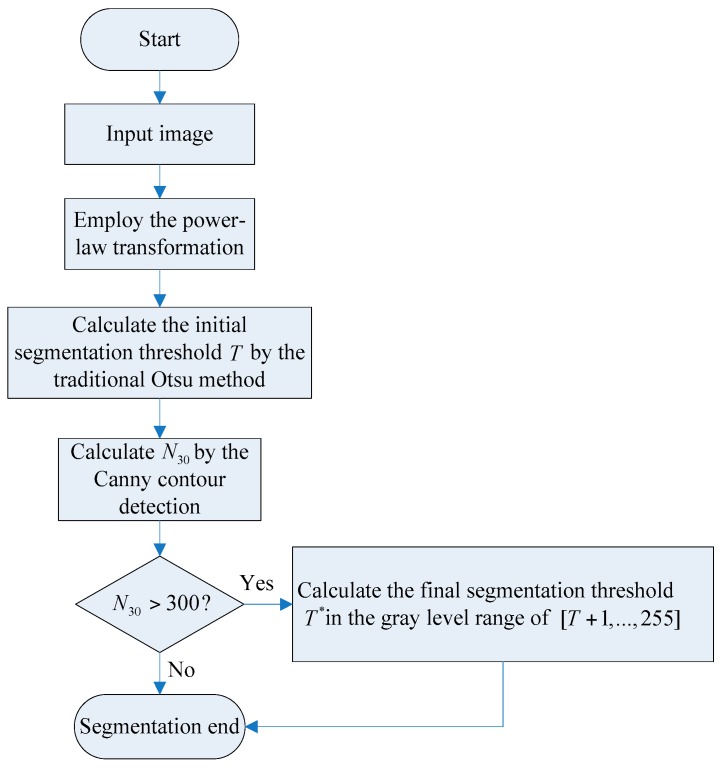
The procedure of the improved Otsu TSM.

When using the traditional TSM to separate the foreground object, in this case a shipwreck, most of the background spots are still retained in the segmentation results. To solve this problem, an improved Otsu TSM is presented that constrains the search range of the ideal segmentation threshold to extract the foreground object inside the image. Since the area size of the background spots, shown in Figure 1a, is usually no bigger than 30 pixels, the parameter *N*_30_ has been defined as the number of contours to be found with an area size smaller than 30 pixels. The procedure of the improved Otsu approach is illustrated in Figure 2. At first, the traditional Otsu method [42] is used to calculate the initial segmentation threshold *T*. Then, the Moore Neighbor contour detection algorithm [43,44] is employed to compute *N*_30_. If *N*_30_ > 300, (64,000/30 × 300 = 71.1:1), it means that there are still many small bright spots remaining in the segmentation result, and the threshold needs to be improved. The final segmentation threshold T* can be calculated as explained further on. If *N*_30_ ≤ 300, the final segmentation threshold T* is set as the initial segmentation threshold *T*, and segmentation is finished. Notice that both values, *N*_30_ and 300 should be changed depending on the characteristics of the used sonar images.

In the gray level range of one plus the initial segmentation threshold *T* calculated by the traditional Otsu method to the gray level of 255, denoted as [*T* + 1,…,255], the number of pixels at gray level *i* is denoted by *n_i_*, and the total number of pixels is calculated by:

(1) $$N=\sum_{i=T+1}^{255}n_{i}$$

The gray level histogram is normalized and regarded as a probability distribution:

(2) $$p_{i}=\frac{n_{i}}{N},p_{i}\geq 0,\sum_{i=T+1}^{255}p_{i}=1$$

Supposing that the pixels are dichotomized into two categories *C*_0_ and *C*_1_ by a threshold T*. The set *C*_0_ implies the background pixels with a gray level of [*T* + 1, …, T*], and *C*_1_ means those pixels of foreground object with a gray level of [*T* + 1, …, 255]. The probabilities of gray level distributions for the two classes are the following: *w*_0_ is the probability of the background and *w*_1_ is the probability of the object:

(3) $$w_{0}=P_{r}(C_{0})=\sum_{i=T+1}^{T^{*}}p_{i},\;w_{1}=P_{r}(C_{1})=\sum_{i=T^{*}+1}^{255}p_{i}=1-w_{0}$$

The means of the two categories *C*_0_ and *C*_1_ are:

(4) $$u_{0}=\sum_{i=T+1}^{T^{*}}ip_{i}/w_{0},\;u_{1}=\sum_{i=T^{*}+1}^{255}ip_{i}/w_{1}$$

The total mean of gray levels is denoted by:

(5) $$u_{M}=w_{0}u_{0}+w_{1}u_{1}$$

The two class variances are given by:

(6) $$\sigma_{0}^{2}=\sum_{i=T+1}^{T^{*}}{\left(i-u_{0}\right)}^{2}p_{i}/w_{0},\;\sigma_{1}^{2}=\sum_{i=T^{*}+1}^{255}{\left(i-u_{1}\right)}^{2}p_{i}/w_{1}$$

The within-class variance is:

(7) $$\sigma_{W}^{2}=w_{0}\sigma_{0}^{2}+w_{1}\sigma_{1}^{2}$$

The between-class variance is:

(8) $$\sigma_{B}^{2}=w_{0}{\left(u_{0}-u_{M}\right)}^{2}+w_{1}{\left(u_{1}-u_{M}\right)}^{2}=w_{0}w_{1}{\left(u_{0}-u_{1}\right)}^{2}$$

The total variance of the gray levels is:

(9) $$\sigma_{M}^{2}=\sigma_{W}^{2}+\sigma_{B}^{2}$$

The final threshold T* is chosen by maximizing the between-class variance, which is equivalent to minimizing the within-class variance, since the total variance, which is the sum of the within-class variance $\sigma_{W}^{2}$ and the between-class variance $\sigma_{B}^{2}$, is constant for different partitions:

(10) $$∴T^{*}=\arg\{\underset{T+1\leq i\leq 255}{\max}\{\sigma_{B}^{2}(i)\}\}=\arg\{\underset{T+1\leq i\leq 255}{\min}\{\sigma_{W}^{2}(i)\}\}$$

### 3.3. The Power-Law Transformation 

**Figure 3:**
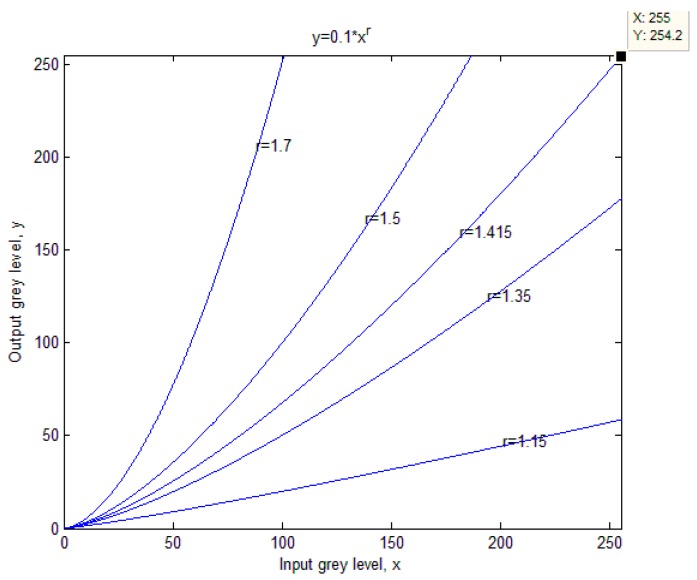
The plots of the power-law equation with different *r* values.

Nevertheless, the Otsu method does have its limitations in that the correct image segmentation cannot be obtained when the gray level of objects is closely approximate to that of the background or the proportion of objects is low. To solve this problem, the gray level for objects should be increased and the one for the background should be decreased for enhancing the contrast and highlighting before using the segmentation method. For this purpose, the power-law transformation has been employed, which has the following basic form:

(11) $$y=cx^{r}$$

where *x* and *y* are the input and output gray levels, *c* and *r* are positive constants. With the help of this power-law transformation, the gray level of each pixel can be easily changed. The plots of this equation with various *r* values are displayed in Figure 3. Different values of *c* and *r* affect the segmentation result directly, many experiments have been done and it has been figured out that the best thresholding result is achieved when the value of *c* is 0.1 and the value of *r* equals 1.415.

### 3.4. TSM Results for Side-Scan Sonar Images

For comparison, the SSS image in Figure 1a,b have been segmented with the traditional Otsu method, the local TSM, the iterative TSM, the maximum entropy TSM and our method, respectively. The local TSM adapts the threshold value on every pixel to the local image characteristics, and a different threshold is selected for each pixel in the image. As for the iterative TSM, it compares the threshold value of each pixel with the average of the maximum and the minimum thresholds in the image. The maximum entropy TSM employs the entropy of the foreground and background regions, the cross-entropy between the original and binarized image, etc. [45].

#### 3.4.1. TSM Results for High Resolution SSS Image

**Figure 4:**
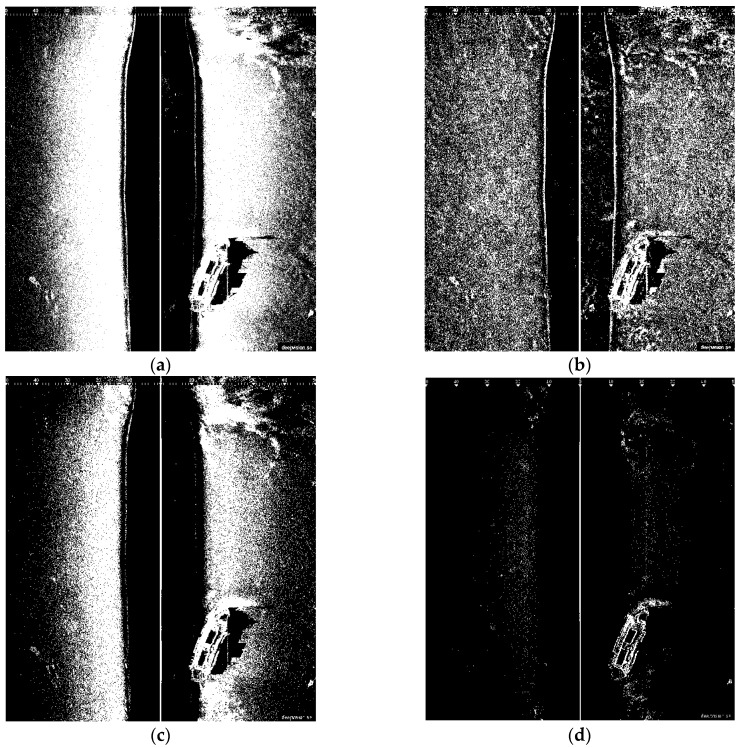
(**a**) Traditional Otsu TSM, *Th* = 0.3216; (**b**) Local TSM, *Th* = 0.1628; (**c**) Iterative TSM, *Th* = 0.4238; (**d**) Maximum entropy TSM, *Th* = 0.6627.

**Figure 5:**
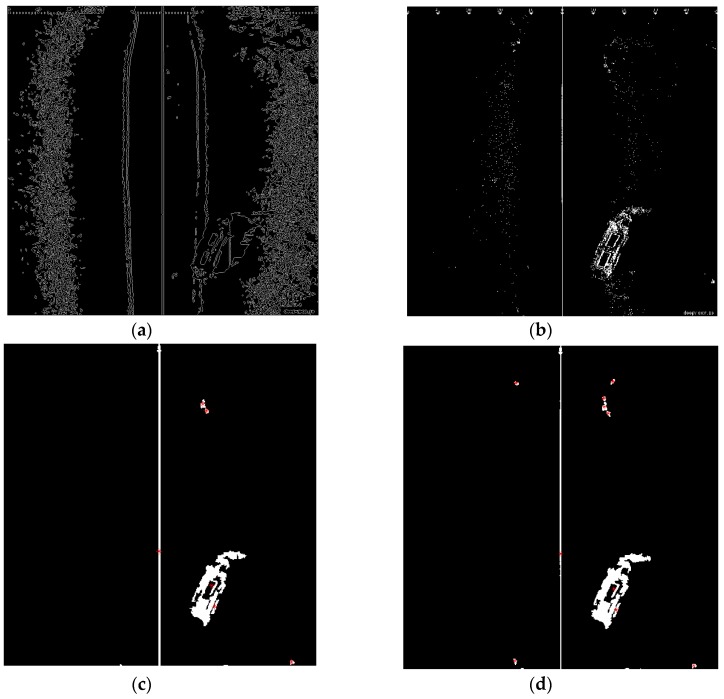
(**a**) Canny edge detection after applying the traditional Otsu method, bw = edge (b, ‘canny’, 0.33), *N*_30_ = 752 > 300; (**b**) Improved Otsu TSM,*T* = 0.3216, T* = 0.6784; (**c**) Result of the improved Otsu TSM after morphological operations marking the centroids of the obtained regions; (**d**) Result of the maximum entropy TSM after the same morphological operations marking the centroids of the acquired areas.

The thresholding segmentation results of the above four classic segmentation methods and the improved Otsu TSM on Figure 1a are shown in Figure 4 and Figure 5, respectively. The output after the TSM operation is a binary image which indicates the object with a gray level of 255 (white pixels in 8-bit images) and the background with a gray level of 0 (black pixels in 8-bit images). Compared with other conventional TSM, it is obvious that the improved Otsu TSM could reduce the influence of noise and also the small bright spots in sonar images, since most of them have been divided into the background. In addition, the segmentation results of the presented method are more precise.

Edges exist between objects in contact, between objects and background and between different fields. As a crucial characteristic of an image, an edge indicates the position of its outline. Consequently, edge detection is the first necessary step in detecting objects and its result directly domains the ability of system recognition and classification [46]. In this paper, the Canny edge detector is used as a feature extraction tool. The main stages of the Canny edge detection algorithm (Algorithm 1) [47] are described as follows, and the result is illustrated in Figure 5a:
**Algorithm 1: Canny edge detection**1. Smooth the image with a Gaussian filter, *h = fspecial (‘gaussian’, [3 3], 0.5)*;2. Calculate the gradient’s amplitude and orientation with the finite-difference for the first partial derivative;3. Non-Maxima Suppression;4. Detect and link the edge with double threshold method, *y = edge (b*, *‘canny’*, *0.33)*, the high threshold for Figure 1a is 0.33, and the 0.4 times high threshold is used for the low threshold.

The initial segmentation threshold *T* computed by the traditional Otsu method is 0.3216, and the parameter *N*_30_ returned from the above Canny edge algorithm equals 752, which is bigger than 300. Therefore, our improved Otsu TSM has been applied, and the segmentation result is shown in Figure 5b, with the final threshold T* of 0.6784. In order to detect the centroids of each segmented region, we need to do the following morphological operations (Algorithm 2) with Figure 5b.
**Algorithm 2: Morphological operations for detecting feature centroids**1. Remove all connected components that have fewer than 30 pixels in Figure 5b;2. Bridge previously unconnnected pixels;3. Perform dilation using the structuring element ones (3) with the size of a 3 × 3 square;4. Fill the holes in the image;5. Compute the area size, the centroid and the bounding box of different contiguous regions;6. Concatenate structure array which contains all centroids into a single matrix.

In Figure 5c, the red stars ‘*’mark the centroids for each contiguous region or connected component in this image. We set the top left corner as the coordinates origin, where the horizontal direction corresponds to the *x*-axis, and the vertical direction to the *y*-axis. The centroid coordinates of all connnected regions within the foreground shipwreck are (535, 603) and (542.3, 653.9), which will be used as point landmarks in the further test of AEKF-based SLAM loop mapping. So the central centroid of this ship is (538.7, 628.5), which is calculated as the average of the above two centroid positions. The same morphological operations for marking the feature centroids is performed on the segmentation result of the maximum entropy TSM, shown in Figure 5d. The confusion matrix of real ship centroids and the ones detected by the improved Otsu TSM is shown in the following Table 1.

Thus, the false positive rate (FPR) of ship centroids detected by the improved Otsu TSM is:

(12) $$FPR=\frac{FP}{FP+TN}=\frac{4}{4+21}=0.16$$

Among several classic segmentation methods compared above, the maximum entropy TSM achieves the best segmentation performances. To compare it here with the proposed method, the following Table 2 shows also the confusion matrix of real ship centroids and the ones detected by the maximum entropy TSM.

In this case, the FPR of ship centroids detected by the maximum entropy TSM is:

(13) $$FPR=\frac{FP}{FP+TN}=\frac{8}{8+20}=0.29$$

which is higher than the FPR of the proposed improved Otsu TSM. As a further performance indicator, the detection precision, also called positive predictive value (PPV), has been calculated for both segmentation methods. For the improved Otsu TSM it is:

(14) $$PPV=\frac{TP}{TP+FP}=\frac{2}{2+4}=0.33$$

while the maximum entropy TSM only leads to a lower value:

(15) $$PPV=\frac{TP}{TP+FP}=\frac{2}{2+8}=0.2$$

Indeed, both precision rates are not really high because the proportion of the foreground ship feature is small, and the centroids in some parts of the background, where their gray levels are similar to that of the ship, are also detected. So, we consider even more important that the improved Otsu TSM shows a better performance and therefore seems to be more robust. The computational cost of the improved Otsu TSM has also been compared with the above mentioned four conventional segmentation approaches, executed on Figure 1a, results are shown in Table 3.

In general, the improved Otsu TSM achieves a more accurate and faster segmentation on the SSS image shown in Figure 1a. Although the computational time of the improved Otsu TSM is 4.9 times higher than that of the classic Otsu method, it is only half of that of the maximum entropy TSM, which achieves the highest segmentation precision among the four classic segmentation methods compared above.

#### 3.4.2. TSM Results for Low Resolution SSS Image

To further compare the performance of the proposed segmentation algorithm with lower resolution images, the same process has been realized over the image shown in Figure 1b. The size of the image is 417 × 228 = 95,076 pixels.Since the background spots, whose gray levels are similar to those of some parts of the object (a branch) in the foreground, usually have an area size not bigger than 15 pixels, the parameter N_15_ has been defined as the number of contours to be found with an area size smaller than 15 pixels. If N_15_ > 100, (95076/15 × 100 = 63.4:1),this assigned threshold of 63.4 is lower than that of 71.1 for Figure 1a, since the proportion of background spots in this low resolution SSS image is higher than that in Figure 1a. 

**Figure 6:**
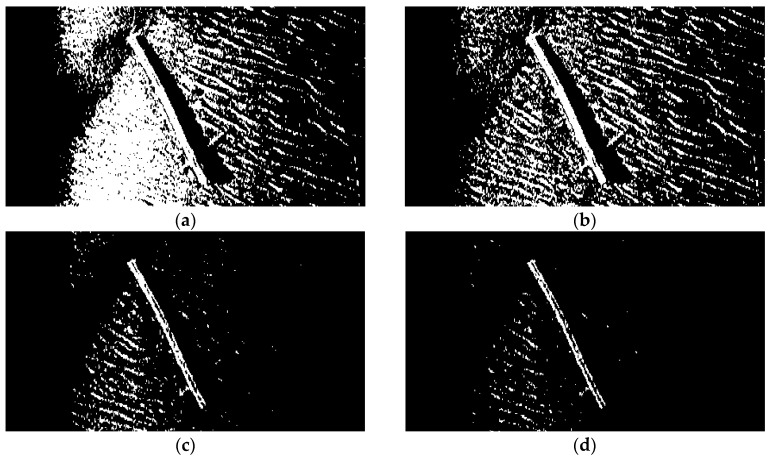
(**a**) Traditional Otsu TSM, *Th* = 0.1137; (**b**) Local TSM, *Th* = 0.0941; (**c**) Iterative TSM, *Th* = 0.2609; (**d**) Maximum entropy TSM, *Th* = 0.3176.

**Figure 7:**
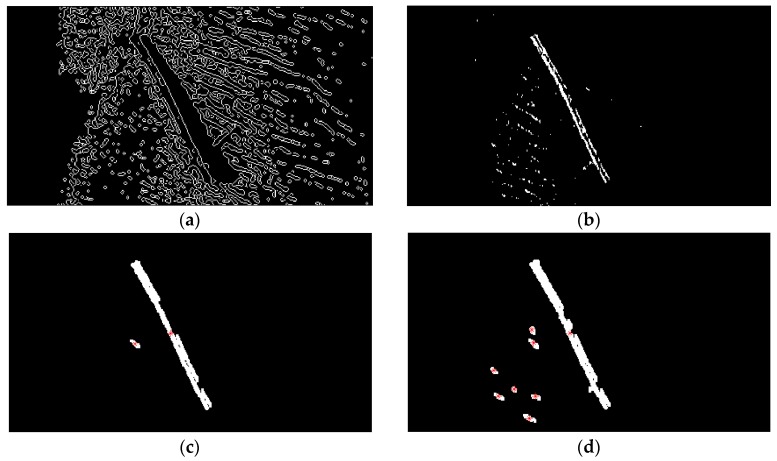
(**a**) Canny contour detection after applying the traditional Otsu method, bw=edge (b, ‘canny’, 0.1255), *N*_15_ = 419 > 100; (**b**) Improved Otsu TSM, *T* = 0.1137, T* = 0.3529; (**c**) Result of the improved Otsu TSM after morphological operations marking the centroids of the obtained regions; (**d**) Result of the maximum entropy TSM after the same morphological operations marking the centroids of the acquired areas.

Also, the lower resolution of the image in Figure 1b and the rough texture of the seabed leads to many small bright spots remaining in the segmentation result, and the threshold needs to be improved. Figure 6 shows the results of several classic segmentation methods and Figure 7b the result of our improved Otsu TSM. The initial segmentation threshold *T* calculated by the traditional Otsu method is 0.1137. In Figure 7a, the parameter *N*_15_ computed by the Canny contour detection algorithm is 419, which is bigger than 100. As a result, the proposed improved Otsu TSM has been applied, and the segmentation result is shown in Figure 7b, with the final threshold T* of 0.3529. The morphological operations for marking the centroids of every segmented region within the branch are similar to that of the ship. Only in step 1, the parameter is set to 15 to remove all connected components that have fewer than 15 pixels. The red stars ‘*’, shown in Figure 7c,d, imply the centroids for every contiguous region or connected component in the segmentation results of our improved Otsu TSM and the maximum entropy TSM, separately. The centroid coordinate of the branch detected by our method is (187.3, 115.6), which will be used as a landmark point in the further simulation test of an AEKF-based SLAM loop mapping. The confusion matrices of the real centroids and the ones detected by the improved Otsu TSM on the one hand and the maximum entropy TSM on the other hand are shown in the following Table 4 and Table 5, separately.

Therefore, the FPR of branch centroids detected by the improved Otsu TSM is:

(16) $$FPR=\frac{FP}{FP+TN}=\frac{1}{1+13}=0.07$$

and the precision of branch centroids detected by the improved Otsu TSM is:

(17) $$PPV=\frac{TP}{TP+FP}=\frac{1}{1+1}=0.5$$

As a result, the FPR of branch centroids detected by the maximum entropy TSM is:

(18) $$FPR=\frac{FP}{FP+TN}=\frac{7}{7+11}=0.39$$

which is 5.5 times bigger than the FPR of the proposed improved Otsu TSM. The precision of branch centroids detected by the maximum entropy TSM is:

(19) $$PPV=\frac{TP}{TP+FP}=\frac{1}{1+8}=0.11$$

which is much lower than the precision of the improved Otsu TSM. As in the previous image, both precision values are low, since the ratio of the foreground branch feature is small, and the centroids of certain parts of the background, where their gray levels are close to those of the branch are also detected, but the difference between precision values is even bigger, 4.5 times higher for the improved Otsu TSM in this image, 1.65 times higher in the formerly presented high resolution image. This proves that the proposed improved Otsu TSM is very valuable, above all for standard sonar images. The computational cost of the proposed improved Otsu TSM is compared with the above four classic segmentation approaches on Figure 1b and is shown in Table 6.

In conclusion, the testing of different SSS images with different resolutions shows that our proposed improved Otsu TSM keeps a good tradeoff between segmentation precision and computational cost. The computational time of the improved Otsu TSM on this low resolution SSS image, shown in Figure 1b, is only two times higher than that of the traditional Otsu TSM. As for the former high resolution SSS image, shown in Figure 1a, it is nearly five times higher. Therefore, the improved Otsu TSM performs even better for lower resolution images both in segmentation precision and processing time and seems to be more robust. In comparison, the configuration-conjunct TSM proposed in [24], which is only suitable for detecting simple linear objects with neat and obvious edges, needs a computing time of 0.371 s on a low resolution sonar image of size 140 × 228 = 31,920 pixels. Executed on that same image, our improved Otsu TSM only spends 0.117 s segmenting the object (a pipe), this means that the configuration-conjunct TSM consumes three times more processor time than our improved Otsu TSM. As a result, the improved Otsu TSM provides a much faster segmentation than other state of the art approaches for detecting underwater objects of different shapes.

### 3.5. TSM Results for Forward-Looking Sonar Image

**Figure 8:**
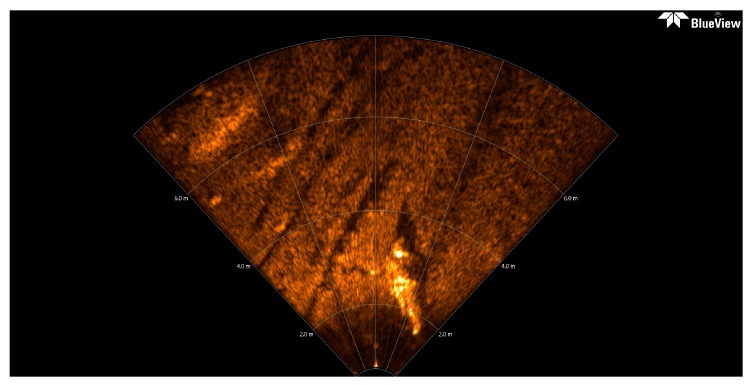
The original FLS image comes from [48], and there is a plastic mannequin in the down center.

Usually, a preliminary mission of AUV is data collection, generally accomplished by means of SSS or multibeam echosounder, another key issue is to ensure the safety of the AUV. For the purpose of avoiding obstacles, the AUV could be equipped with a forward-looking sonar (FLS) to sense the working environment at a certain distance in the direction of navigation. The FLS platform emits a short acoustic pulse in forward direction on a horizontal sector of around 120°, and on a vertical sector from 15° to 20°. The original FLS imagery used here (see Figure 8) is provided by Desistek Robotik Elektronik Yazilim company (Ankara, Turkey) [48]. It is recorded with the Blue View P900-90 2D Imaging Sonar, which has a 90° horizontal field of view, and works at a frequency of 900 kHz. Its update rates are up to 15 Hz, the scanning range is 100 m, and its resolution is 2.51 cm.

The size of the converted FLS imagery is 1920 × 932 = 1,789,440 pixels, the area size of the background spots is usually fewer than 40 pixels, with gray levels similar to those of certain areas of the foreground objects. In this case, *N*_40_ is defined as the number of contours which area size is smaller than 40 pixels, and it is computed by the Canny contour detection algorithm. If *N*_40_ > 600, (1,789,440/40 × 600 = 74.6:1), this assigned threshold 74.6 is higher than that of 71.1 for the former high resolution SSS image of the ship, that is because the black background proportion in this presented FLS image is higher than that in that SSS image. This means that there are many small bright spots still left in the segmentation result. 

**Figure 9:**
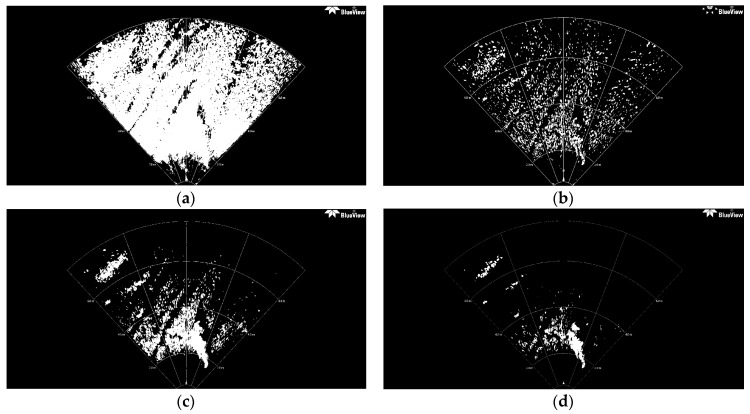
(**a**) Traditional Otsu TSM, *Th* = 0.1176; (**b**) Local TSM, *Th* = 0.0941; (**c**) Iterative TSM, *Th* = 0.2990; (**d**) Maximum entropy TSM, *Th* = 0.4118.

**Figure 10:**
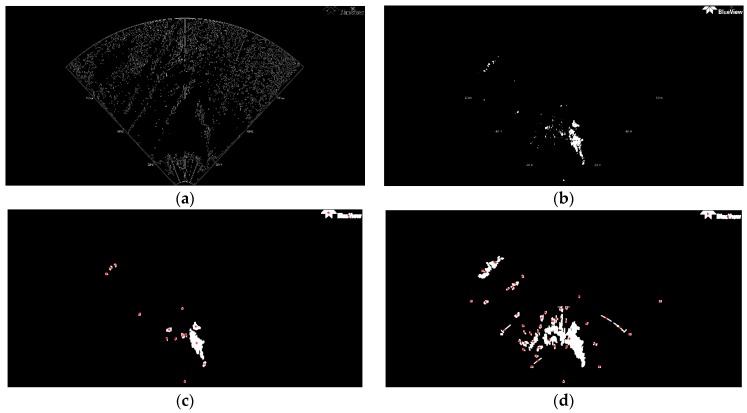
(**a**) Canny edge detection after employing the traditional Otsu method, bw = edge (b, ‘canny’, 0.13), *N*_40_ = 1341 > 600; (**b**) Improved Otsu TSM, *T* = 0.1176, T* = 0.5412; (**c**) Result of the improved Otsu TSM after morphological operations marking the centroids of the acquired areas; (**d**) Result of the maximum entropy TSM after the same morphological operations marking the centroids of the obtained regions.

The initial FLS image in Figure 8 has been segmented with the traditional Otsu method, the local TSM, the iterative TSM, the maximum entropy TSM and our method, respectively. The results of these segmentations are shown in Figure 9 and Figure 10.The initial segmentation threshold *T* obtained via the traditional Otsu method is 0.1176. In Figure 10a, the parameter *N*_40_ calculated from the above Canny edge detection algorithm is 1341, which is bigger than 600. Thus, our improved Otsu TSM has been applied, and the segmentation result is shown in Figure 10b, with the final threshold T* of 0.5412. The morphological operations for computing the centroids of every segmented region within the body are similar to that of the ship. Only in step 1, the parameter is set to 40, in order to remove all connected components that have fewer than 40 pixels. Besides, in step 3, it applies dilation two times. 

The red stars ‘*’, shown in Figure 10c, stand for the centroids for each contiguous region or connected component in this image. The centroid coordinates of every connected region within the foreground object are (949.8, 662.9), (966.9, 660.6), (1021.2, 615.7), (1024.1, 703) and (1065.5, 811.3), and will be used as landmark points in the further simulation test of an AEKF-based SLAM loop mapping. So, the center centroid of the body is (1005.5, 690.7), which is calculated as the average of the above five centroids. The same morphological operations for marking the feature centroids is employed on the segmentation result of the maximum entropy TSM, shown in Figure 10d. The confusion matrices of the real body centroids and the ones detected by the improved Otsu TSM on the one hand and the maximum entropy TSM on the other hand are shown in the following Table 7 and Table 8, respectively.

As for the former SSS images, we calculate the FPR and PPV indicators:

(20) $$FPR=\frac{FP}{FP+TN}=\frac{11}{11+26}=0.297$$

(21) $$PPV=\frac{TP}{TP+FP}=\frac{5}{5+11}=0.3125$$

In this case, the FPR and the precision value are:

(22) $$FPR=\frac{FP}{FP+TN}=\frac{44}{44+40}=0.524$$

(23) $$PPV=\frac{TP}{TP+FP}=\frac{2}{2+44}=0.043$$

As can be seen, also for the FLS images, similar performance is achieved with the improved Otsu TSM. The FPR value is 1.8 times lower and the precision is seven times higher, even better than for the SSS images, which means that, the lower the image quality is, the better the detection performance for important features. Besides, three real body centroids are not detected at all by the maximum entropy TSM. In general, in all SSS and FLS images presented in this work, the proposed improved Otsu TSM has much lower FPR and much higher precision rate on the detected feature centroids than those values of the maximum entropy TSM. 

Finally, again the computational cost of the proposed improved Otsu TSM is compared with the above indicated four conventional segmentation methods on executed Figure 8 and the results are shown in Table 9. It is higher than for the traditional Otsu TSM, the local TSM and the iterative TSM, but nearly one third of the time needed by the maximum entropy TSM, Instead, much better detection rates are achieved.

In general, the improved Otsu TSM constrains the search range of the ideal segmentation threshold, and combined with the contour detection algorithm, the foreground object of interest, a ship, a branch and a body have been separated more accurately, in sonar images of very different resolutions and qualities, with a low computational time. Compared with the maximum entropy TSM, which has the highest segmentation accuracy among the four conventional segmentation approaches compared above, our improved Otsu TSM just needs half of the processing time for segmenting the ship in the high resolution SSS image. Regarding the branch in the low resolution SSS image, our method consumes two thirds of the time, and for the body in the presented FLS image, it only spends one third of the computational time used by the maximum entropy TSM. As a result, the improved Otsu TSM achieves precise threshold segmentation performances for underwater feature detection at a fast computational speed. Since the computational time of the improved Otsu TSM is very short, it achieves real time results which can be used afterwards for underwater SLAM. The centroids that have been calculated for the different objects will be used as landmark points in the following AEKF-SLAM loop map simulation. Moreover, compared to the traditional Otsu method, the presented approach could retain more complete information and details of objects after segmentation, also the holes and gaps in objects are reduced.

## 4. The Estimation-Theoretic AEKF-SLAM Approach

The mathematical framework employed in the study of the AEKF-based underwater landmark SLAM is reviewed in this section. This framework is identical in all respects to that applied in Smith et al. [5] and uses the same notation as that adopted in Leonard and Durrant-Whyte [49]. Since features may look totally different from different directions, this paper uses the least complicated features, which are stationary point landmarks. This simplification not only reduces challenges with feature identification and interpretation, it also increases the focus on the AEKF-SLAM algorithm itself.

### 4.1. Extended Kalman Filter

In the SLAM problem, KF or EKF is employed to produce estimates of the robot pose and landmark positions. If the SLAM problem is linear and Gaussian, then the conventional KF is guaranteed to converge. However, real-world SLAM applications are usually nonlinear or even discontinuous (as in the presented underwater scenario), a standard approach when dealing with nonlinear system is to first linearize the system around the current state and then use an EKF. Although the linearization errors caused by the standard EKF lowers the performance of the localization estimate, EKF still tends to provide satisfactory results in general. Therefore, EKF is often regarded as the “gold standard” of comparison for online SLAM algorithms due to its conceptual simplicity, high convergence, relatively low computational cost and capacity to handle uncertainty. The nonlinear discontinuous system is assumed to have the following form:

(24) $$\text{State function }f(\cdot ):\;X_{k}=f(X_{k-1})+\varpi_{k}$$

(25) $$\text{Observation function }h(\cdot ):\;Z_{k}=h(X_{k})+v_{k}$$

where $\varpi_{k}$ is the process noise and complies with standard normal distribution $\varpi_{k}\sim N(0,Q_{k})$, its covariance matrix is defined as *Q_k_*; *v_k_* is the observation noise and it also obeys standard Gaussian distribution $v_{k}\sim N(0,R_{k})$, its covariance matrix is denoted as *R_k_*.

1. Time Update
  - Predictor step:
    (26) $${\hat{X}}_{k}^{-}=f({\hat{X}}_{k-1}^{-})$$
    (27) $$P_{k}^{-}=F_{k}P_{k-1}F_{k}^{T}+Q_{k}$$
    where ${\hat{X}}_{k}^{-}$ is the system’s current state vector, and $P_{k}^{-}$ is its covariance matrix. *F_k_* and *H_k_* are the Jacobian matrices of partial derivatives of f(⋅) and h(⋅) with respect to *X*.
    (28) $$F_{k}={\frac{\partial f(X)}{\partial X}|}_{X={\hat{X}}_{k-1}^{-}},\;H_{k}={\frac{\partial h(X)}{\partial X}|}_{X={\hat{X}}_{k}^{-}}$$
    The nonlinear functions *f* and h are linearized by using a Taylor series expansion, where terms of second and higher order are omitted.
2. Measurement Update
  • Calculate the Kalman gain *K_k_*, $K_{k}=P_{k}^{-}H_{k}^{T}{\left(H_{k}P_{k}^{-}H_{k}^{T}+R_{k}\right)}^{-1}$.
  • Corrector step:
    First, update the expected value ${\hat{X}}_{k}$, ${\hat{X}}_{k}={\hat{X}}_{k}^{-}+K_{k}[Z_{k}-h({\hat{X}}_{k}^{-})]$.
    Then, update the error covariance matrix $P_{k}$, $P_{k}=P_{k}^{-}-K_{k}H_{k}P_{k}^{-}=(I-K_{k}H_{k})P_{k}^{-}$.

### 4.2. The Estimation Process of the AEKF-SLAM

As can be found in [50], the root mean square (RMS) error in AEKF-SLAM just before loop closure is much smaller than the one in the EKF-SLAM. Also, the AEKF-SLAM can compensate the odometric error of the robot more exactly and generate a more precise feature map than the EKF-SLAM. As for FastSLAM [51], which uses Rao-Blackwellised method for particle filtering (RBPF), it is better than EKF-SLAM at solving the data association problem for detecting loop closures, and it is suitable for non-linear motions. Nevertheless, the biased noises arising from unequal wheel diameters and wheel misalignment skew the robot’s odometric trajectory to one side and conventional SLAM methods, such as EKF/Fast SLAM cannot guarantee good performance in this situation, since they assume zero mean noise when correcting odometric error. Particle filters work best in grid constrained areas, such as corridors, and in the representation of occupancy grid maps, not in landmark maps like those considered in this work. Another challenge in the particle filtering SLAM is to reduce the number of particles while maintaining the estimation accuracy. For indoor environments and aerial structures (such as roads or buildings), particle filters are well suited. However, in sonar applications there are usually no such constraints. Therefore, the AEKF method is chosen to solve the SLAM problem in this paper.

**Figure 11:**
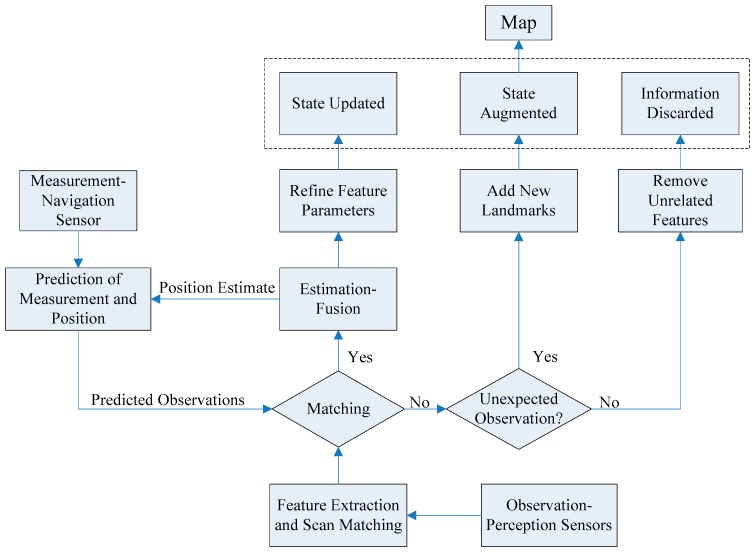
The flow chart of SLAM procedure based on an AEKF. Modified after [27].

The AEKF-based SLAM approach is a recursive and iterative estimation-update process, which besides a prediction and an update stage (as in classical EKF), includes an augmentation stage. The prediction stage is to reckon the current state of the robot with its on-board navigation sensors. The observation stage is to measure the features by the imaging sensor and to obtain the relative positions to the robot. Then, the relative positions of detected landmarks are transformed into the robot’s coordinates system. The next matching involves a data association process, where landmarks from the predicted and observed maps are matched together and their differences are used to update the landmark positions and refine the estimated robot pose. The measurements from the sonar image include three kinds of information: new features, associated features and unrelated information. After data association, new features will augment the state vector, associated features can improve the precise degree of the state, and offensive features are discarded directly. The whole procedure of the AEKF-SLAM algorithm is depicted in Figure 11.

In the prediction stage, the command signal u_k_ and the robot motion model are utilized to estimate the robot pose. Then, in the update stage, the innovation v_k_ is computed as the difference between the new observation from an exteroceptive sensor and the predicted measurement, and its error covariance is used to calculate the Kalman gain W_k_. When a landmark is detected for the first time, it is added to the system state vector through the state augmentation stage.

**Figure 12:**
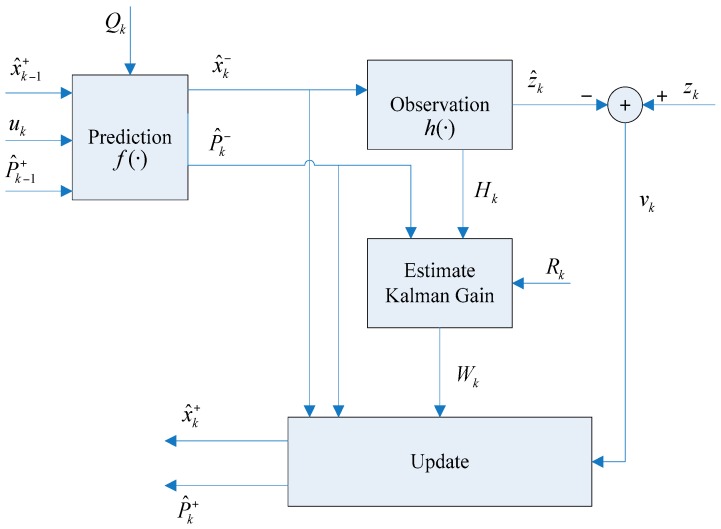
The architecture of the AEKF-SLAM system, as described in [50].

Figure 12 illustrates the architecture of the AEKF-SLAM system. Let ${\hat{X}}_{k}$ and ${\hat{P}}_{k}$ be the estimated state vector and covariance. Then the filter recursively updates the mean ${\hat{X}}_{k}^{+}$ and covariance ${\hat{P}}_{k}^{+}$ of the state by combining the predicted mean ${\hat{X}}_{k}^{-}$ and covariance ${\hat{P}}_{k}^{-}$ with the new observation *z_k_*. Here *Q_k_* and *R_k_* are the covariance matrices of procession noise errors and observation errors, respectively. 

The pseudo code of the AEKF-based SLAM algorithm (Algorithm 3) for underwater landmark map is outlined as follows, where *z_f_* is the vector of landmarks that are already detected and stored in the map; *z_n_* is the vector of measurements which are unseen and new landmarks.
**Algorithm 3**: Underwater landmark map building based on AEKF-SLAM1. For k=1 to N2. $[X_{k}^{-},P_{k}^{-}]=Predict(X_{k-1},P_{k-1});$3. $z_{k}=Get\;Observations\;();$4. $[z_{f},z_{n}]=Data\;Association(X_{k}^{-},P_{k}^{-},z_{k},R_{k});$5. $[X_{k}^{+},P_{k}^{+}]=Update\;Map(X_{k}^{-},P_{k}^{-},z_{f},R_{k});$6. $[X_{k}^{+},P_{k}^{+}]=Augment\;Map(X_{k}^{-},P_{k}^{-},z_{n},R_{k});$7. End for

We assume that the actual 3D space geometry is orthogonal to the horizontal plane in which the robot moves, so that the world can be adequately represented by a 2D model. The fundamental equations for the landmark map SLAM based on the AEKF are presented in the following. Detailed descriptions can be found in [50]. 

#### 4.2.1. Prediction Stage

The AEKF-based SLAM map is defined by an extended state vector ${\hat{X}}_{a}$, which is composed of the current robot pose${\hat{X}}_{v}$ and all observed environmental landmark positions ${\hat{X}}_{m}$ [52]. Here *P_vm_* stands for the cross covariance between the robot state and the map landmarks:

(29) $${\hat{X}}_{a}=[\begin{array}{c} {\hat{X}}_{v}\\ {\hat{X}}_{m} \end{array}],\;P_{a}=[\begin{array}{cc} P_{v} & P_{vm}\\ P_{vm}^{T} & P_{m} \end{array}]$$

The robot state ${\hat{X}}_{v}$ is represented by its position and heading angle vectors in Cartesian coordinates as ${\hat{X}}_{v}={\left[{\hat{x}}_{v},{\hat{y}}_{v},{\hat{\varphi }}_{v}\right]}^{T}$, and its covariance matrix is *P_v_*. Supposing the position of the *n*-th landmark is denoted as $x_{m_{n}}={\left({\hat{x}}_{n},{\hat{y}}_{n}\right)}^{T}$, and the environmental landmarks are described as ${\hat{X}}_{m}={\left[{\hat{x}}_{1},{\hat{y}}_{1},...,{\hat{x}}_{n},{\hat{y}}_{n}\right]}^{T}$, and its covariance matrix is *P_m_*. Note that the initial condition of the state estimate is usually given as ${\hat{X}}_{a}={\hat{X}}_{v}=0$ and *P_a_* = *P_v_* = 0, which means that no landmarks have been observed yet and the initial robot pose defines the base coordinate origin.

An estimate of the robot pose change ${\hat{X}}_{\delta }={\left[{\hat{x}}_{\delta },{\hat{y}}_{\delta },{\hat{\varphi }}_{\delta }\right]}^{T}$ with covariance *P_δ_* is commonly obtained using wheel encoder odometry and a robot kinematic model. Therefore, the prediction state of the system is given by:

(30) $${\hat{X}}_{a}^{-}=f({\hat{X}}_{a},{\hat{X}}_{\delta })=[\begin{array}{c} g({\hat{X}}_{v},{\hat{X}}_{\delta })\\ {\hat{X}}_{m} \end{array}]=[\begin{array}{c} {\hat{x}}_{v}+{\hat{x}}_{\delta }\cos{\hat{\varphi }}_{v}-{\hat{y}}_{\delta }\sin{\hat{\varphi }}_{v}\\ {\hat{y}}_{v}+{\hat{x}}_{\delta }\sin{\hat{\varphi }}_{v}+{\hat{y}}_{\delta }\cos{\hat{\varphi }}_{v}\\ {\hat{\varphi }}_{v}+{\hat{\varphi }}_{\delta }\\ {\hat{X}}_{m} \end{array}]$$

and its prediction covariance matrix is:

(31) $$P_{a}^{-}=JP_{a}J^{T}+QP_{\delta }Q^{T}$$

where the Jacobian matrices *J* and *Q* are:

(32) $$J={\frac{\partial f}{\partial X_{a}}|}_{\left({\hat{X}}_{a},{\hat{X}}_{\delta }\right)}={[\begin{array}{cc} J_{v} & 0_{vm}\\ 0_{vm}^{T} & I_{m} \end{array}]}_{\left({\hat{X}}_{a},{\hat{X}}_{\delta }\right)},\;Q={\frac{\partial f}{\partial X_{\delta }}|}_{\left({\hat{X}}_{a},{\hat{X}}_{\delta }\right)}={[\begin{array}{c} Q_{v}\\ 0_{vm}^{T} \end{array}]}_{\left({\hat{X}}_{a},{\hat{X}}_{\delta }\right)}$$

where *J_v_* and *Q_v_* are the Jacobian matrices of partial derivatives of the nonlinear model function *g* with respect to the robot state *X_v_* and the robot pose change *X_δ_*:

(33) $$J_{v}={\frac{\partial g}{\partial X_{v}}|}_{\left({\hat{X}}_{v},{\hat{X}}_{\delta }\right)}=[\begin{array}{ccc} 1 & 0 & -{\hat{x}}_{\delta }\sin{\hat{\varphi }}_{v}-{\hat{y}}_{\delta }\cos{\hat{\varphi }}_{v}\\ 0 & 1 & {\hat{x}}_{\delta }\cos{\hat{\varphi }}_{v}-{\hat{y}}_{\delta }\sin{\hat{\varphi }}_{v}\\ 0 & 0 & 1 \end{array}],\;Q_{v}={\frac{\partial g}{\partial X_{\delta }}|}_{\left({\hat{X}}_{v},{\hat{X}}_{\delta }\right)}=[\begin{array}{ccc} \cos{\hat{\varphi }}_{v} & -\sin{\hat{\varphi }}_{v} & 0\\ \sin{\hat{\varphi }}_{v} & \cos{\hat{\varphi }}_{v} & 0\\ 0 & 0 & 1 \end{array}]$$

Since these Jacobians only affect the robot portion of the covariance matrix *P_v_* and its cross-correlations *P_vm_*, the prediction covariance matrix $P_{a}^{-}$ can be implemented more efficiently as:

(34) $$∴P_{a}^{-}=[\begin{array}{cc} J_{v}P_{v}J_{v}^{T}+Q_{v}P_{\delta }Q_{v}^{T} & J_{v}P_{vm}\\ {\left(J_{v}P_{vm}\right)}^{T} & P_{m} \end{array}]$$

#### 4.2.2. Update Stage

Suppose that a feature already stored in the map as an estimate $x_{m_{i}}={\left({\hat{x}}_{i},{\hat{y}}_{i}\right)}^{T}$ is observed by a range-bearing sonar with the measurement *z*:

(35) $$z=[\begin{array}{c} r\\ \theta \end{array}],\;R=[\begin{array}{cc} \sigma_{r}^{2} & \sigma_{r\theta }^{2}\\ \sigma_{r\theta }^{2} & \sigma_{\theta }^{2} \end{array}]$$

where (*r,θ*) denotes the distance and the orientation of the detected landmark with respect to the robot, and *R* is the observation covariance. 

Next, transform the sensed position value from the global coordinates to the robot coordinates using the following transformation. With this correlation to the robot pose estimate, different map landmarks can be associated with each other, and these correlations increase monotonically until their relative to each other positions become perfectly known:

(36) $${\hat{z}}_{i}=h_{i}({\hat{X}}_{a}^{-})=[\begin{array}{c} \sqrt{{\left({\hat{x}}_{i}-{\hat{x}}_{v}\right)}^{2}+{\left({\hat{y}}_{i}-{\hat{y}}_{v}\right)}^{2}}\\ \arctan(\frac{{\hat{y}}_{i}-{\hat{y}}_{v}}{{\hat{x}}_{i}-{\hat{x}}_{v}})-{\hat{\varphi }}_{v} \end{array}]$$

If the observation *z* is associated with the estimated map landmark ${\left({\hat{x}}_{i},{\hat{y}}_{i}\right)}^{T}$ correctly, then the SLAM results are updated:

(37) $${\hat{X}}_{a}^{+}={\hat{X}}_{a}^{-}+W_{i}v_{i}$$

(38) $$P_{a}^{+}=P_{a}^{-}-W_{i}S_{i}W_{i}^{T}$$

The update process starts with computing the measurements residual *v_i_*, also called innovation:

(39) $$v_{i}=z-h_{i}({\hat{X}}_{a}^{-})$$

which is the difference between the actual observed and predicted measurements. Its covariance matrix *S_i_* is:

(40) $$S_{i}=HP_{a}^{-}H^{T}+R$$

and the Kalman gain *W_i_*:

(41) $$W_{i}=P_{a}^{-}H^{T}S_{i}^{-1}$$

where *H* represents the Jacobian matrix which linearizes the nonlinear measurements function *h* around the best estimation of the state ${\hat{X}}_{a}^{-}$.

#### 4.2.3. State Augmentation

As the environment is explored, newly detected landmarks must be added to the stored map. Here, a method for initializing new features is provided. First, the state vector and covariance matrix are augmented with the new observation *z_new_* and its covariance matrix *R_new_*, which are measured relative to the robot:

(42) $${\hat{X}}_{aug}=[\begin{array}{c} {\hat{X}}_{a}\\ z_{new} \end{array}],\;P_{aug}=[\begin{array}{ccc} P_{v} & P_{vm} & 0\\ P_{vm}^{T} & P_{m} & 0\\ 0 & 0 & R_{new} \end{array}]$$

The extension state can be initialized to the correct values by a linearized transformation *f_i_*, which is defined as follows: the transformation function *g_i_* is applied to convert the polar observation *z_new_* to a global Cartesian feature position. It is composed of the current robot pose ${\hat{X}}_{v}$ and the new observation *z_new_*:

(43) $${\hat{X}}_{a}^{+}=f_{i}({\hat{X}}_{aug})=[\begin{array}{c} {\hat{X}}_{a}\\ g_{i}({\hat{X}}_{v},z_{new}) \end{array}]=[\begin{array}{c} {\hat{X}}_{a}\\ x_{v}+r\cos(\theta +{\hat{\varphi }}_{v})\\ y_{v}+r\sin(\theta +{\hat{\varphi }}_{v}) \end{array}]$$

(44) $$P_{a}^{+}=\nabla f_{x_{aug}}P_{aug}\nabla f_{x_{aug}}^{T}$$

where the sparse Jacobian matrix $\nabla f_{x_{aug}}$ is given by:

(45) $$\nabla f_{x_{aug}}={\frac{\partial f_{i}}{\partial X_{aug}}|}_{{\hat{X}}_{aug}}=[\begin{array}{ccc} I_{v} & 0 & 0\\ 0 & I_{m} & 0\\ G_{X_{v}} & 0 & G_{z_{new}} \end{array}]$$

and the Jacobian matrices $G_{X_{v}}$ and $G_{Z_{new}}$ are:

(46) $$G_{X_{v}}={\frac{\partial g_{i}}{\partial X_{v}}|}_{\left({\hat{X}}_{v},z_{new}\right)}=[\begin{array}{ccc} 1 & 0 & -r\sin(\theta +{\hat{\varphi }}_{v})\\ 0 & 1 & r\cos(\theta +{\hat{\varphi }}_{v}) \end{array}],\;G_{Z_{new}}={\frac{\partial g_{i}}{\partial Z_{new}}|}_{\left({\hat{X}}_{v},z_{new}\right)}\;=[\begin{array}{cc} \cos(\theta +{\hat{\varphi }}_{v}) & -r\sin(\theta +{\hat{\varphi }}_{v})\\ \sin(\theta +{\hat{\varphi }}_{v}) & r\cos(\theta +{\hat{\varphi }}_{v}) \end{array}]$$

The matrix multiplication of $P_{a}^{+}$ requires $O(n^{3})$ computation complexity where *n* is the number of landmarks on the map. Due to the sparseness of the Jacobian matrix, a much more efficient transformation can be implemented. It only affects the block diagonal matrix of the newly observed landmark and off diagonal cross-correlations to the rest of the map:

(47) $$∴P_{a}^{+}=[\begin{array}{ccc} P_{v} & P_{vm} & P_{v}G_{X_{v}}^{T}\\ P_{vm}^{T} & P_{m} & P_{vm}^{T}G_{X_{v}}^{T}\\ G_{X_{v}}P_{v} & G_{X_{v}}P_{vm} & G_{X_{v}}P_{v}G_{X_{v}}^{T}+G_{z_{new}}R_{new}G_{z_{new}}^{T} \end{array}]$$

### 4.3. AEKF-SLAM Loop Map Simulation 

The following MATLAB simulation experiment is carried out for an AEKF-based SLAM loop mapping, which is performed in the context of a generic mobile robot observing the surrounding point landmarks (including not only the calculated centroids of the shipwreck, branch and plastic mannequin, but also those centroids of certain parts of the background detected by the formerly presented improved Otsu TSM in Section 3) with a range-bearing sensor in a two-dimensional area. The values of different parameters could be changed according to the number of detected landmarks and their positions in the environment, the actual speed of the robot, and the maximum observation distance of the selected sonar sensor. 

**Figure 13:**
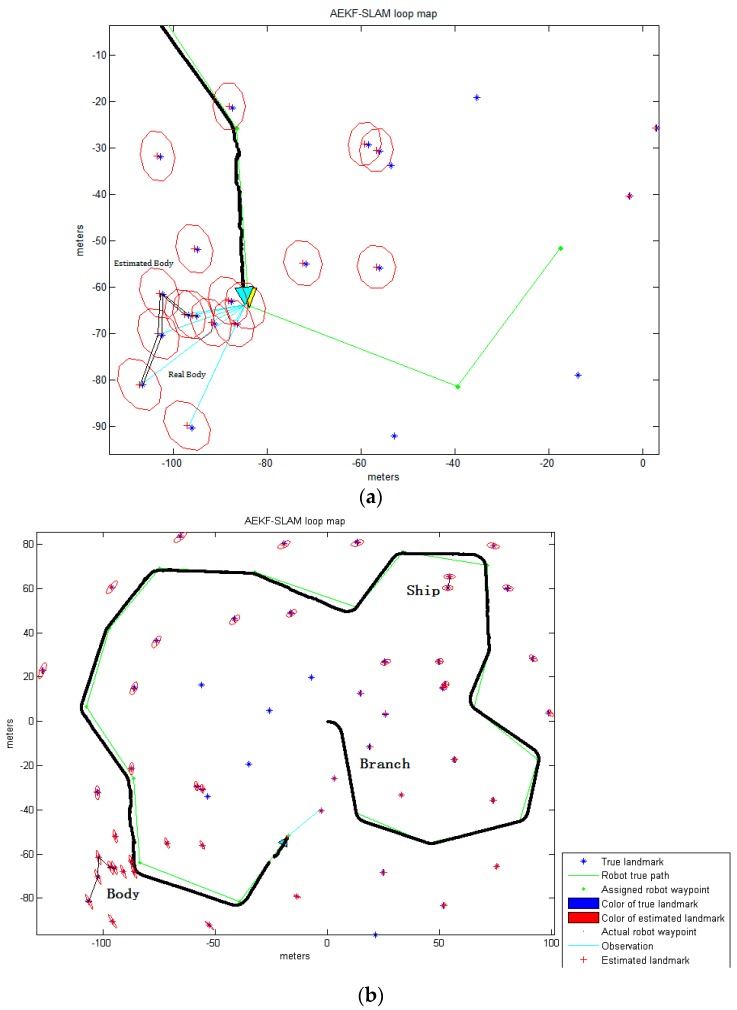
(**a**) The robot is observing the centroids of certain parts of the body before loop closure; (**b**) The final AEKF-SLAM loop map where the landmarks are detected by the improved Otsu TSM.

The simulation environment for the AEKF-SLAM loop map, where the landmarks are detected by the improved Otsu TSM is defined as an area of 200 m ×200 m, containing 17 robot waypoints connected as a circle and 54 landmarks distributed near the robot path, as shown in Figure 13. Since the environment is an area of 200 m × 200 m (from −100 m to 100 m), we set the centroid coordinates of the ship as (53.5, 60.3), (54.23, 65.39), the body centroids as (−94.98, −66.29), (−96.69, −66.06), (−102.12, −61.57), (−102.41, −70.3), (−106.55, −81.13) and the branch centroid as (18.7, −11.6). Besides, those centroids of certain parts of the background detected by the improved Otsu TSM are also included. The velocity of the robot has been fixed to 3 m/s, its deviation to 0.3 m/s, and the steering angle error to 3π/180 rad. The range and bearing of observation variance is 0.1 m/s and π/180 rad, respectively. The sampling time is 0.0125 s and the robot measures an observation each 0.1 s, which means that the robot gets an image each 0.3 m. The maximum observation distance is 30 m.

Figure 13 demonstrates the 2D landmark map obtained by the AEKF-based SLAM algorithm, where the landmarks are detected by the improved Otsu TSM. The positions of sensor scans for the point landmarks are clearly visible, and few of these sightings are rejected based on statistical outlier rejection techniques [53], since they are out of the observation range of the robot. The rest are believed to represent a landmark and are added into the map. The true landmarks are shown as blue stars ‘*’, green dots ‘⋅’ are the robot waypoints which are used for calculating the steering angle, and the red crosses ‘+’ are the estimated landmark positions. The ellipses around each red cross illustrate the uncertainty covariances for the landmark coordinate estimations. The estimated robot path is depicted as the solid black line, leaded by a cyan triangle. Around this cyan triangle we can see a red ellipse, which stands for the covariance of the AEKF estimate projected to the robot pose. The larger it is, the more uncertain the current robot pose is. The perceptions are drawn as cyan lines, in Figure 13a, the robot is observing the centroids of some parts of the body. After navigation, the robot has identified the centroid positions of different portions of the ship, branch and body, which are connected by the black line in Figure 13b.

The landmark point positions of the ship, branch and body estimated by the AEKF and the real ones detected by the improved Otsu TSM are shown in Table 10.

Therefore, the standard deviation between landmark point positions of different features estimated by the AEKF and the true ones detected by the improved Otsu TSM is:

(48) $$\sigma_{1}=0.124$$

**Figure 14:**
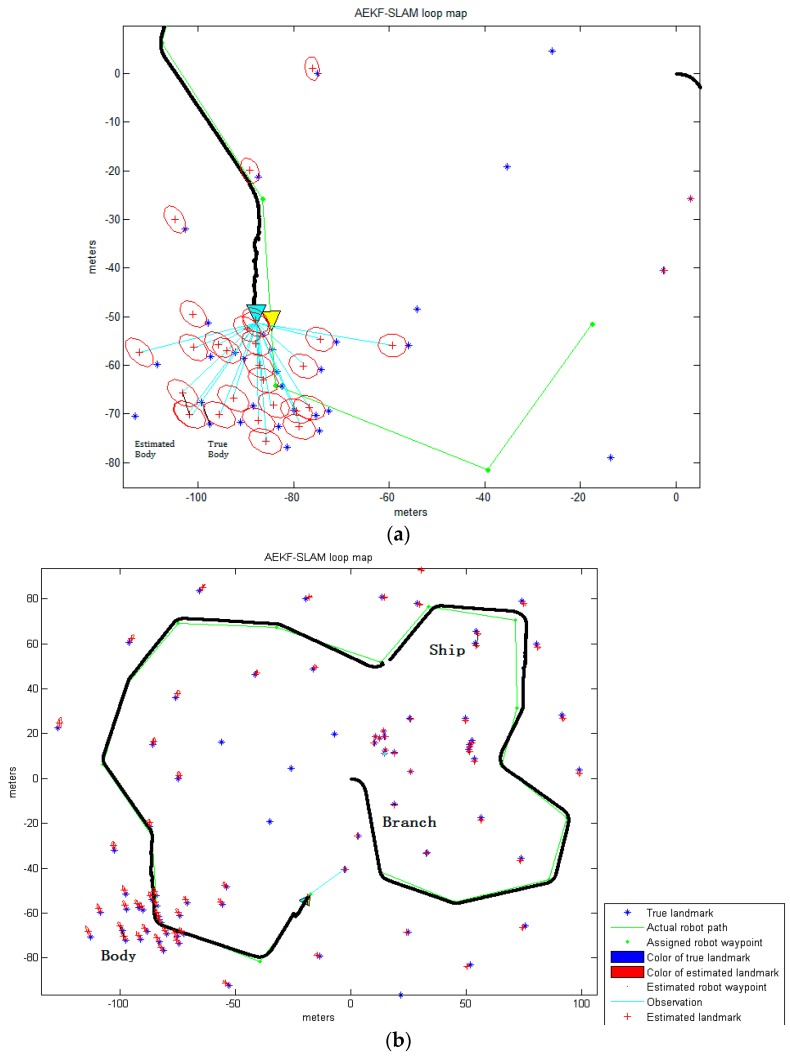
(**a**) The robot is observing the centroids of certain parts of the body before loop closure; (**b**) The final AEKF-SLAM loop map where the landmarks are detected by the maximum entropy TSM.

For comparison, the AEKF-SLAM loop map where 77 landmarks are detected by the maximum entropy TSM is shown in Figure 14. 

The observations of the body parts are shown in Figure 14a, while Figure 14b shows, as before, the corresponding final estimated robot path after navigation. Table 11 contains the data for the landmark point positions of the ship, branch and body estimated by the AEKF and the real ones detected by the maximum entropy TSM.

As a result, the standard deviation between landmark point positions of different features estimated by the AEKF and the real ones detected by the maximum entropy TSM is:

(49) $$\sigma_{2}=1.37$$

which is much larger than that of the improved Otsu TSM. Thus, the AEKF achieves more accuracy and estimates the robot pose more robustly due to the landmark positions obtained by our improved Otsu TSM, than those detected by the maximum entropy TSM. Although the maximum entropy TSM achieves the highest segmentation precision over the other three classic segmentation methods compared above, it has much lower precision than our improved Otsu TSM, and it also needs much more computation time. Moreover, it can be found that since the position of the first observed landmark point is known with high accuracy, the uncertainty in the estimated robot pose will decrease significantly when the robot completes the loop and revisits this landmark. Thus, in turn, the uncertainties of previously detected landmarks are reduced. Also, the AEKF-based SLAM algorithm achieves reliable detection of cycles in the map and consistent map update on loop closure.

## 5. Conclusions and Future Work

### 5.1. Conclusions

In this work, first a detailed state of the art of approaches for the underwater SLAM problem has been presented, the three most commonly used map representations are also compared, and in our case, the landmark map is chosen to represent the underwater exploring region. Besides, the improved iterative Otsu TSM constrains the search range of the ideal segmentation threshold. Combined with the Canny contour detection algorithm, the foreground objects of interest (a shipwreck from a high resolution SSS image, a branch from a low resolution SSS image and a plastic mannequin from a FLS image) have been separated more accurately than with other conventional TSMs like the traditional Otsu method, the local TSM, the iterative TSM and the maximum entropy TSM. Compared with the traditional Otsu method, the presented method could keep more complete information and details of objects after segmentation, also the holes and gaps in objects are reduced. What’s more, the computational cost of our proposed improved Otsu TSM is much lower compared to the maximum entropy TSM, which has the highest segmentation accuracy of the four classic methods. Moreover, the improved Otsu TSM has much lower FPR and much higher precision rate on the detected feature centroids than those values of the maximum entropy TSM in all SSS and FLS images presented in this work. In conclusion, the improved Otsu TSM achieves much more precise and fast segmentation performances than other methods for detecting underwater objects of different shapes.

Afterwards, in order to verify the proposed segmentation method, the simulation of an AEKF solution for the underwater SLAM navigation is performed using both, the centroids of every part of the ship, the branch and the body, plus those centroids of certain parts of the background as landmark points. After navigation, the robot has localized the centroids of different segments of the ship, branch and body as point landmarks. Here, the AEKF has achieved more accuracy and more robust estimations of the robot pose and the landmark positions, using the centroids detected by our improved Otsu TSM than with those detected by the maximum entropy TSM. Also, the AEKF-SLAM has achieved reliable detection of cycles in the map and consistent map update on loop closure. Furthermore, the MATLAB simulation experiment for AEKF-SLAM loop mapping has shown excellent performance in map management in terms of landmark addition and removal to avoid the long-term accumulation of clutter in the map. 

### 5.2. Future Work

In the next step, with the aim of the “Smart and Networking Underwater Robots in Cooperation Meshes”-SWARMs project, our proposed improved Otsu segmentation method will be tested and validated with the help of the AEKF to the underwater SLAM problem in subsea scenarios in the Atlantic Ocean, the Black Sea and Norwegian coastline with extreme sea water conditions in terms of sea low temperatures (1–3 °C), high currents (<1 m/s), low visibility (<1 m), high pressures (<15 atm), and 1000–2000 m^2^ of working area. As for further improvements of the current study, the future work includes:

• Employing other forms of target objects for the detection and tracking purpose, devising parametric feature models for describing general objects, and more complex scenarios with multiple distinct features will also be included. Besides, more complicated vehicle model such as six DOF kinematic model will be investigated. Therefore, as the robot navigates, we can perform the proposed feature detection algorithm on the acquired images exactly when the 3D object is detected by the sonar.
• Developing a computationally tractable version of the SLAM map building algorithm which maintains the properties of being consistent and non-divergent. Hierarchical SLAM or sub-mapping methods build local maps of limited size, which bound the covariances and thus the linearization errors. Then, by linking the local maps through a global map or a hierarchy of global maps, AEKF-based SLAM application in large environments is possible.
• Considering the application of unscented KF (UKF) in the field of underwater robotic navigation. As an alternative estimation technique, UKF does not need calculating the derivatives, and it also handles a very effective tradeoff between computational load and estimation accuracy in the case of strongly nonlinear and discontinuous systems [54]. Besides, considering FastSLAM, which uses the Rao-Blackwellised method for particle filtering (RBPF), as future work, since it is very suitable for non-linear motions. It also has better performance than EKF-SLAM at solving the data association problem for detecting loop closures. Afterwards, we will evaluate the estimation performances of these two methods to the SLAM problem with that of the AEKF considered in this work.
• Incorporating data streams observed from the acoustic and visual sensors to generate a 3D representation of the underwater environment, i.e., the seabed, working environment or artifacts [55]. In our case, we will use the depth logger based on pressure for navigation and the DE340D SSS as perception sensor to get horizontal positions of features of interest, therefore by integrating with the vertical positioning data obtained through pressure sensor, a subsea 3D map will be created.
• Considering map simplification and transform based algorithms for fusion of two different resolution maps. One is a large scale medium resolution map generated using a SSS (in SWARMs T4.1 Large scale 3D mapping), the other is a local 3D high resolution map created by fusion of FLS images and visual information. The sonar system used to obtain the large scale map achieves a very high area coverage rate but has a modest resolution, as it could detect objects but is insufficient to identify their precise nature. To achieve combining both systems for maximizing the operational effectiveness, the large scale medium resolution map will be used to trigger detailed investigations of regions of interest using the local 3D high resolution maps.

## Figures and Tables

**Table 1:** The confusion matrix of real and detected ship centroids using the improved Otsu TSM.

		Detected	
		Ship Centroids	Non-Ship Centroids
**Real**	**Ship Centroids**	2	0
	**Non-Ship Centroids**	4	21

**Table 2:** The confusion matrix of real and detected ship centroids using the maximum entropy TSM.

		Detected	
		Ship Centroids	Non-Ship Centroids
**Real**	**Ship Centroids**	2	0
	**Non-Ship Centroids**	8	20

**Table 3:** Computational costs of different segmentation methods on Figure 1a.

Segmentation Method	Computational Time [s]
Traditional Otsu TSM	0.178226
Local TSM	0.913942
Iterative TSM	0.289513
Maximum entropy TSM	1.562499
**Improved Otsu TSM**	**0.868372**

**Table 4:** The confusion matrix of real and detected branch centroids using the improved Otsu TSM.

		Detected	
		Branch Centroids	Non-Branch Centroids
**Real**	**Branch Centroids**	1	0
	**Non-Branch Centroids**	1	13

**Table 5:** The confusion matrix of real and detected branch centroids using the maximum entropy TSM.

		Detected	
		Branch Centroids	Non-Branch Centroids
**Real**	**Branch Centroids**	1	0
	**Non-Branch Centroids**	7	11

**Table 6:** Computational costs of different segmentation methods on Figure 1b.

Segmentation Method	Computational Time [s]
Traditional Otsu TSM	0.120458
Local TSM	0.261021
Iterative TSM	0.227290
Maximum entropy TSM	0.378283
**Improved Otsu TSM**	**0.241164**

**Table 7:** The confusion matrix of real and detected body centroids using the improved Otsu TSM.

		Detected	
		Body Centroids	Non-Body Centroids
**Real**	**Body Centroids**	5	0
	**Non-Body Centroids**	11	26

**Table 8:** The confusion matrix of real and detected body centroids using the maximum entropy TSM.

		Detected	
		Body Centroids	Non-Body Centroids
**Real**	**Body Centroids**	2	3
	**Non-Body Centroids**	44	40

**Table 9:** Computational costs of different segmentation methods on Figure 8.

Segmentation Method	Computational Time [s]
Traditional Otsu TSM	0.244472
Local TSM	0.941853
Iterative TSM	0.428126
Maximum entropy TSM	3.903889
**Improved Otsu TSM**	**1.452562**

**Table 10:** The landmark point positions of the ship, branch and body estimated by the AEKF and the true ones detected by the improved Otsu TSM.

	Ship [m]		Branch [m]	Body [m]				
True	(53.5, 60.3)	(54.23, 65.39)	(18.73, −11.56)	(−94.98, −66.29)	(−96.69, −66.06)	(−102.12, −61.57)	(−102.41, −70.3)	(−106.55, −81.13)
Estimated	(53.66, 60.23)	(54.31, 65.32)	(18.8, −11.49)	(−94.99, −66.35)	(−96.67, −66.12)	(−102.2, −61.59)	(−102.4, −70.34)	(−106.4, −81.44)

**Table 11:** The landmark point positions of the ship, branch and body estimated by the AEKF and the true ones detected by the maximum entropy TSM.

	Ship [m]		Branch [m]	Body [m]	
True	(53.61, 60.18)	(54.22, 65.4)	(18.75, −11.55)	(−99.23, −67.7)	(−97.59, −72.08)
Estimated	(54.24, 59.12)	(54.96, 64.35)	(18.62, −11.69)	(−100.1, −65.82)	(−98.58, −70.23)

## Abbreviations

The following abbreviations are used in this manuscript:

SONAR	SOund Navigation And Ranging
SLAM	Simultaneous Localization and Mapping
TSM	Threshold Segmentation Method
SSS	Side-Scan Sonar
FLS	Forward-Looking Sonar
AEKF	Augmented Extended Kalman Filter
KF	Kalman Filter
EKF	Extended Kalman Filter
PF	Particle Filter
EM	Expectation Maximization
SIFT	Scale-Invariant Feature Transform
SURF	Speeded Up Robust Features
DOF	Degree of Freedom
CML	Concurrent Mapping and Localization
RSSI	Received Signal Strength Indication
AUV	Autonomous Underwater Vehicle
FRR	False Positive Rate
PPV	Positive Predictive Value
RMS	Root Mean Square
RBPF	Rao-Blackwellised Particle Filtering
UKF	Unscented Kalman Filter
